# Adipose Tissue, Natriuretic Peptides, and HFpEF: Clinical Implications of the Obesity Paradox

**DOI:** 10.3390/biomedicines14061305

**Published:** 2026-06-09

**Authors:** Michał Maksymilian Wilk, Piotr Gajewski

**Affiliations:** Institute of Heart Diseases, Wroclaw Medical University, 50-367 Wroclaw, Poland; dr.piotr.gajewski@gmail.com

**Keywords:** HFpEF, obesity, adipose tissue, natriuretic peptides

## Abstract

**Introduction**: Adipose tissue (AT) is increasingly recognized as an active endocrine and immunological organ involved in the pathophysiology of heart failure with preserved ejection fraction (HFpEF). Dysfunctional AT, particularly visceral, promotes chronic low-grade inflammation, endothelial dysfunction, and microvascular damage. At the same time, higher body mass is associated with paradoxically lower natriuretic peptide (NP) levels, which may impact diagnostic accuracy in HFpEF. **Methods**: This narrative review summarizes the current evidence on the interplay between adipose tissue, NPs, and HFpEF, focusing on pathophysiological mechanisms, AT distribution, and clinical implications. **Results**: Adipokine-mediated inflammation contributes to the myocardial stiffness, fibrosis, and cardiac remodeling characteristic of HFpEF. Visceral adipose tissue, including epicardial fat, exhibits a more proinflammatory profile than subcutaneous fat. Obesity is associated with decreased NP levels due to increased clearance and decreased production. Consequently, lower NP levels may lead to underdiagnosis or misclassification of HFpEF, particularly in diagnostic algorithms such as HFA-PEFF and H_2_FPEF. Patients with low BMI or cachexia exhibit elevated NP levels, reflecting advanced disease and catabolic states. **Conclusions**: The obesity-natriuretic paradox poses a key diagnostic challenge in HFpEF. Interpretation of natriuretic peptide levels should take body composition into account, and refinement of biomarker cutoff values may improve diagnostic accuracy.

## 1. Introduction

Recently, much attention has been paid to the role of adipose tissue (AT) in the context of heart failure with preserved ejection fraction (HFpEF), especially an approach where AT is presented as an active endocrine and immunological organ [1,2]. Specifically, this is because of the rich profile of secreted mediators called adipokines, which can directly influence the function of the cardiovascular system [3]. According to the so-called “adipokine hypothesis” proposed by Milton Packer, adipokines produced by dysfunctional (visceral) AT may induce chronic low-grade inflammation, leading to vascular endothelial dysfunction, microcirculation damage, and the development of the common metabolic HFpEF phenotype [3,4,5,6,7]. At the same time, some studies have shown a correlation between lower concentrations of natriuretic peptides (NPs) in people with increasing body mass index (BMI), despite greater hemodynamic burden and the risk of developing heart failure (HF) [8]. The described phenomenon, so-called the obesity-natriuretic paradox, may also significantly influence the diagnostic and prognostic assessment in patients with HFpEF [9], as the “optimal” natriuretic peptide cutoff value for detection of HF is still a matter of debate [10,11]. In this review, we discuss the relationships between AT, HFpEF pathophysiology, and observable NP concentrations, taking into account the clinical significance and diagnostic challenges. Additionally, we highlight currently available treatment strategies for the metabolic subtype of HFpEF, taking future prospects into account.

## 2. Methods

A narrative search of the literature was conducted using the keywords “HFpEF,” “obesity,” “adipose tissue,” and “natriuretic peptides.” Relevant studies were identified using major scientific databases such as Pubmed, and Google Scholar. The search of the literature was conducted between January and March 2026. To ensure the relevance and contemporary character of the review, priority was given to publications from 2020 onward. The review primarily relied on original research articles published in high-impact cardiology journals, particularly ESC Heart Failure, Journal of the American College of Cardiology, and European Journal of Heart Failure. Particular emphasis was put on original clinical studies, translational research, scientific statements, and landmark review articles relevant to the topic. Exclusion criteria included studies published before 2020, articles in languages other than English, conference abstracts without full text, and studies not directly relevant to HFpEF, obesity, or natriuretic peptides. Studies with insufficient methodological description, limited relevance to cardiovascular disease, or duplicated findings were also excluded. The final selection of studies was based on their relevance to the pathophysiology, diagnosis, and therapeutic implications of obesity-related HFpEF. This review was designed as a narrative review intended to summarize and discuss current evidence regarding adipose tissue, natriuretic peptides, and HFpEF. Therefore, no formal systematic review protocol, such as PRISMA flowchart, or standardized quality assessment tool was applied.

## 3. Adipose Tissue as a Driver of HFpEF

**Figure 1:**
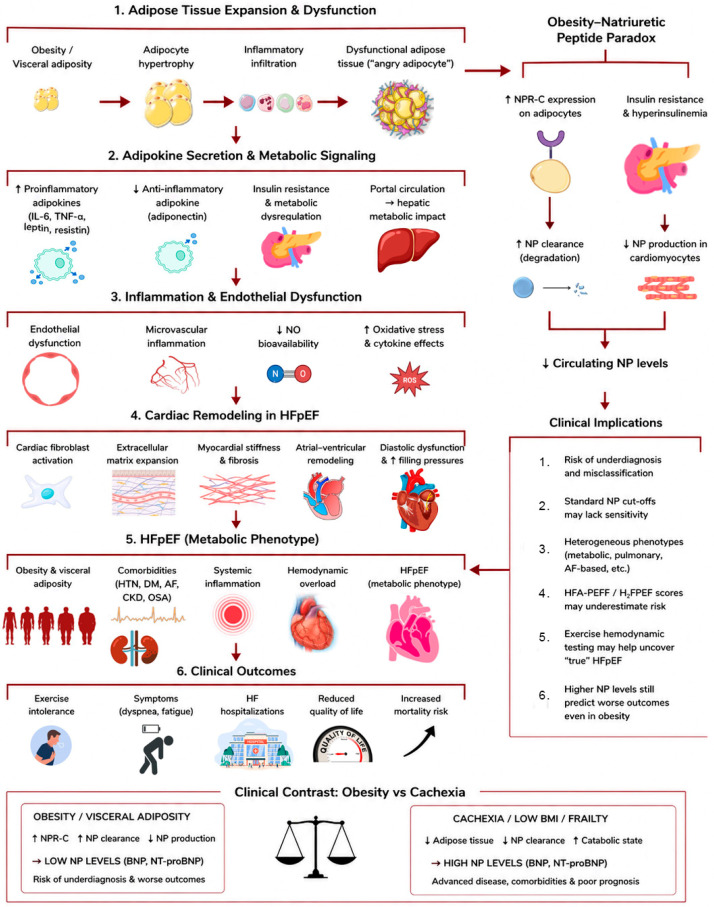
Schematic overview of the proposed pathophysiological mechanisms underlying HFpEF progression in obesity, illustrating the interactions between adipose tissue, systemic inflammation, myocardial remodeling, and their clinical implications.

The paradigm of AT as passive energy storage has been rejected in favor of the view that it is an active endocrine and immunological organ capable of secreting numerous mediators that influence the cardiovascular system [12,13,14]. During body weight gain, adipocyte hypertrophy, ischemic injury, and subsequent inflammatory infiltration, particularly by macrophages, occur [1]. This process promotes chronic low-grade inflammation, which alters the adipokine profile secreted by adipocytes [3,6]. Among these mediators, of particular attention are proinflammatory cytokines such as interleukin 6 (IL-6) and tumor necrosis factor alpha (TNF-α), as well as hormones, most notably leptin and resistin [4]. The mentioned substances can affect neighboring cells, spreading inflammation locally, but they can also cause systemic effects on the cardiovascular system by influencing metabolic maladaptation [4]. The importance of these mechanisms in the pathophysiology of HFpEF has been underscored by the adipokine hypothesis, which posits that adipokines derived from dysfunctional adipose tissue can induce endothelial dysfunction and inflammatory changes in the myocardial microcirculation [4,12]. This process also results in reduced nitric oxide bioavailability and increased myocardial stiffness occurring as a consequence of the activation of cardiac fibroblasts and the subsequent expansion of the extracellular matrix [4,12,14,15]. If this process is prolonged, it leads to cardiac muscle fibrosis and atrio-ventricular remodeling, which further deepens the diastolic dysfunction and pressure overload characteristic of HFpEF [15,16]. Importantly, not all AT-derived mediators exert detrimental cardiovascular effects. Recent studies suggest that brown AT may also produce cardioprotective lipokines capable of improving systemic metabolism, reducing inflammation, and potentially attenuating adverse cardiac remodeling, highlighting the complex and heterogeneous biological role of AT in HFpEF pathophysiology [17,18]. As a result, AT plays a crucial role in the development and course of the metabolic phenotype of HFpEF [19]. The low-grade inflammation is thus not only a pathophysiological mechanism of HF progression but is also associated with poor outcomes [20,21,22]. The described pathophysiology is demonstrated in Figure 1. We also emphasized that further research is needed to understand the impact of AT on the development and progression of HFpEF across different subgroups such as sex and age cohorts [23]. For instance, sex-related differences may additionally influence the interaction between AT and NP biology, because women generally demonstrate higher circulating NP concentrations despite frequently presenting with greater adiposity and higher prevalence of HFpEF [24]. Moreover, sex-specific differences in adipokine profiles AT distribution may contribute to distinct HFpEF phenotypes and potentially influence diagnostic thresholds and therapeutic responses. Given that adipose tissue has become one of the most intriguing therapeutic targets in HF, as well as in the broad population of cardio-renal–metabolic syndrome [25,26,27,28,29,30].

## 4. Visceral Fat Versus Subcutaneous Adipose Tissue

Although BMI is commonly used to define obesity, current data indicate that the subtype and distribution of AT are more important than its total amount in the pathophysiological context [19]. Especially visceral adipose tissue (VAT), which is characterized by a significantly more proinflammatory and metabolically active secretory profile than that described for subcutaneous adipose tissue (SAT) [12,19]. VAT secretes much more IL-6, TNF-α, and resistin than SAT, while significantly less adiponectin, which has anti-inflammatory and insulin-sensitizing effects [16,31]. Additionally, metabolic products and inflammatory mediators of VAT-derived may directly impact hepatic metabolism via portal circulation, exacerbating insulin resistance and systemic inflammation [16,32]. Interestingly, metabolic disturbances and increased cardiovascular risk appear to be primarily related to VAT accumulation rather than to classic SAT obesity, which is often assessed solely by BMI, omitting the important aspect of AT type rather than quantity alone [33,34]. It is worth paying special attention to epicardial adipose tissue (EAT), a subtype of VAT that lies in direct contact with the myocardium and coronary arteries [16,31,33,35]. Importantly, EAT is not separated from the myocardium by a fascial layer, which facilitates direct molecular exchange between these tissues. This proximity allows for paracrine effects of adipokines on cardiac structures [33,36]. From a clinical perspective, EAT can be assessed using several imaging modalities, including echocardiography, computed tomography (CT), and cardiac magnetic resonance imaging (CMR). While echocardiography remains the most accessible technique in daily practice, CT and CMR provide more accurate volumetric quantification and characterization of EAT distribution and inflammatory activity. Therefore, multimodal EAT imaging may represent an important bridge between mechanistic understanding and clinical phenotyping of HFpEF [37]. In addition to paracrine signaling, EAT may also exert vasocrine effects by releasing bioactive mediators directly into the coronary circulation [38]. Accordingly, it is hypothesized that the mentioned EAT activity may influence the cardiac metabolism itself, which may pose a potential therapeutic target in HF [39,40]. In obesity, EAT may undergo hypertrophy and inflammatory infiltrations, which ultimately leads to fibrosis, remodeling, exacerbation of diastolic dysfunction via external pressure on the ventricular wall, and lastly, poorer prognosis in patients with HFpEF [16,36,41]. Therefore, VAT and its specialized form EAT appear to be important factors in the pathogenesis of the metabolic phenotype of HFpEF [16,31]. Moreover, AT could also play an active role in the synthesis and metabolism of several hormones, including aldosterone and its metabolites [42]. This may further contribute to neurohormonal activation, linking AT dysfunction with the vicious cycle of the renin–angiotensin–aldosterone system in HF, however this needs further investigation.

Lastly, given the limitations of BMI in reflecting true AT distribution, alternative body composition assessment tools may provide more clinically meaningful information in patients with suspected HFpEF. Parameters such as waist-to-hip ratio, waist circumference, dual-energy X-ray absorptiometry, or CT-derived VAT area may better characterize metabolically adverse fat distribution and potentially improve the interpretation of NP concentrations in obese individuals [43].

## 5. The Obesity–Natriuretic-Peptide Paradox

Natriuretic peptides (NPs), particularly B-type Natriuretic Peptide (BNP) and its inactive metabolite N-terminal pro-brain natriuretic peptide (NT-proBNP), are useful tools in the diagnosis and risk stratification of patients with HF [8,9,44]. They are secreted by cardiomyocytes in response to pressure overload and the associated ventricular wall stress; therefore, they directly reflect hemodynamic stress and the severity of cardiac dysfunction [9]. Paradoxically, many studies indicate that obese people have significantly lower NP concentrations compared to people with normal body weight or even NPs within the normal range, despite having a higher risk of HF and an unfavorable hemodynamic profile [8,45]. This phenomenon, known as the obesity–natriuretic paradox, has not been fully explained, but some researchers suggest an increased clearance of NPs via AT [46]. Adipocytes are characterized by high expression of C-type natriuretic receptors (NPR-C), which are responsible for the uptake and degradation of circulating NPs [9,46]. As adipose tissue expands, the number of these receptors also increases, resulting in the augmented removal of NPs from the circulation, ultimately resulting in their reduced serum levels with increasing BMI [8,46]. Additionally, insulin resistance and associated hyperinsulinemia, which also characterize excess weight, may play a significant role as well [41,42]. In this case, insulin may modify NPR-C expression through receptor upregulation but also inhibit natriuretic peptide production in cardiomyocytes themselves by altering cellular energy pathways [46,47]. Ultimately, metabolic disorders occurring in obesity may act on NPs at two levels—increasing their clearance and reducing their production, which explains the observed decreasing tendency of these peptides in patients with excess body weight [8,46].

## 6. Clinical Implications of the Obese HFpEF Phenotype

Low NP concentration in patients with obesity has significant clinical meaning, especially in the context of HFpEF diagnosis, for which NPs are one of the basic criteria used to confirm onset of the disease [9]. This is because, as previously mentioned, NPs reflect the degree of hemodynamic overload expressed as cardiac/intravascular wall stretching [9,44]. In the general population, NP values below established cut-off points for different clinical scenarios have a high ability to exclude HF [9]. However, in patients with obesity, reduced NP concentrations may lead to underestimation or incorrect assessment of disease severity, or even its oversight [48]. The likelihood of diagnostic errors in the context of HFpEF is further increased by the heterogeneity of the clinical picture (metabolic, pulmonary, AF-based phenotypes, etc.), in which different components may dominate, overlap, or combine to form complex symptom constellations and distorted test results [9,49]. Importantly, HFpEF may also develop in non-metabolic clinical settings, including structural and congenital heart disease [50]. For example, patients after surgical correction of aortic coarctation have been shown to exhibit an increased long-term risk of HFpEF despite the absence of classic obesity-related mechanisms [50]. This observation further highlights the heterogeneity of HFpEF phenotypes and suggests that natriuretic peptide interpretation may also remain challenging in AT-independent forms of the disease.

Among the well-recognized HFpEF phenotypes is the AF-associated subtype. Importantly, AF itself is a known factor associated with increased NP concentrations; therefore, in patients with obesity-related HFpEF and coexisting AF, the opposing effects of obesity-related NP suppression and AF-related NP elevation may partially counterbalance each other, potentially resulting in biomarker values that do not accurately reflect the severity of the patient’s clinical condition [51]. Regarding the diagnostic algorithms, it is also worth adding the problematic use of the HFA-PEFF and H_2_FPEF scales, which are partially based on the concentration of NPs and BMI value in assessing the probability of HFpEF in a patient with dyspnea [30,52]. Thus, exercise hemodynamic test may help identify “true” HFpEF patients in that case [53]. Reduced concentration of NPs or lower BMI, defined as low SAT with abundant VAT, may result in an underestimation of disease probability and misclassification of the patient into a lower risk category than the actual one [52]. In the case of the HFA-PEFF scale, there are separate cut-off points for NPs depending on whether the patient’s rhythm is AF or sinus rhythm. Additionally, Taylor et al. evaluated age-adjusted NT-proBNP thresholds, suggesting that further refinement of biomarker cut-off values, taking into account factors such as obesity, may improve diagnostic accuracy in HF [11]. By analogy, creating similar calibrations of the scales in relation to parameters reflecting the advancement of obesity—less accurate BMI or a better waist–hip ratio—would help increase the sensitivity of HFpEF screening scales, but this would have to be confirmed and validated in further studies [44]. Moreover, worth noting is the fact that despite the reduced NP concentrations in the case of the metabolic phenotype, they retain significant prognostic utility, as higher values have been shown to be associated with an increased risk of HF hospitalization and mortality within this subgroup [8]. Therefore, proposing separate cut-off points for this clinical scenario might improve diagnostic accuracy [44,54]. At the same time, the importance of a comprehensive clinical evaluation, including imaging tests, structural body assessment, invasive hemodynamic tests, and consideration of comorbidities, is emphasized [30,49,53]. Accordingly, from a practical perspective, in obese patients with suspected HFpEF and disproportionately low NP concentrations, we indicate additional diagnostic modalities such as echocardiographic assessment of diastolic dysfunction, exercise hemodynamic testing, or imaging-based evaluation of VAT and EAT. This may help confirm the diagnosis and improve identification of “true” HFpEF cases despite apparently reassuring biomarker values.

## 7. Clinical Contrast Between Obesity and Cachexia

On the opposite side of the weight profile, there are patients with frailty, sarcopenia and cachexia. HF patients are prone to developing changes in their body composition, and those changes may be responsible for the progression of the disease and its symptoms [22,55,56,57]. Unlike obesity, patients with low body weight often demonstrate significantly elevated NP concentrations, with the highest values among the group with BMI below 18.5 kg/m^2^ [54]. This phenomenon is often due to the advanced morbidity, malnutrition, increased myocardial remodeling, or even presence of other chronic conditions such as cancer or chronic obstructive pulmonary disease, which additionally worsen treatment options and lower survival rates [32,58,59,60]. Additionally, loss of AT may lead to decreased NP clearance, because the decrease in AT volume is also associated with a decrease in the density of NPR-C receptors expressed on adipocytes, potentially promoting their higher concentrations [46]. Furthermore, some researchers point primarily to a correlation between the decrease in axial muscle mass and the increase in NPs, and indicate that this correlation is stronger in HFpEF patients than in those with HF with reduced ejection fraction [61]. In this context, elevated NP levels can be attributed to catabolic systemic alterations during the prolonged disease process [32,62]. Consequently, the relationship between body weight and NP concentration in HF is an inverse relationship, contrasting the natriuretic paradox observed in obesity [32,54,61]. This highlights the complex interactions between AT metabolism and the natriuretic peptide system [4,12]. In the clinical context, this demonstrates the need to interpret NP concentrations in light of the patient’s metabolic phenotype and body composition, as both obesity and cachexia can significantly affect these biomarker levels and their diagnostic and prognostic value [32,44].

## 8. Therapeutic Implications in the Metabolic HFpEF Phenotype

Given the central role of adipose tissue in the pathophysiology of HFpEF, therapeutic strategies targeting obesity and a range of metabolic disorders have recently gained importance [63,64,65]. Weight loss achieved through structured lifestyle interventions, such as dietary modification or physical activity, is often associated with improved exercise capacity and quality of life [66,67]. However, in many cases, particularly in individuals with advanced disease or significant comorbidities, lifestyle interventions alone are insufficient and may require adjunctive pharmacotherapy. In recent years, pharmacotherapy for weight loss has been enriched by two groups of promising drugs. The first of them is glucagon-like peptide-1 (GLP-1) receptor agonists (GLP-1RAs), which have demonstrated significant effects in reducing body weight, improving cardiometabolic parameters, and exerting insulin-sensitizing and anti-inflammatory effects [64,65,68]. These drugs have the potential to directly impact cardiac structure and function by inhibiting inflammation mediated by maladaptive AT, which is also associated with improved metabolic homeostasis [69]. Interestingly, recent pair-feeding studies on animal models suggest that some beneficial cardiovascular effects of GLP-1 receptor agonists may occur independently of weight reduction itself, supporting the hypothesis of direct myocardial and metabolic effects beyond systemic improvement alone [70]. Similarly, researchers are currently focusing on a familiar group of drugs which exert dual incretin effects via the GLP-1 receptor and a second receptor, known as the glucose-dependent insulinotropic peptide (GIP) receptor, which seems to exert an even more pronounced effect than sole GLP-1RAs [71,72]. These medications are actively being studied for their potential role in modifying the metabolic course of HFpEF, which may prove effective by modifying and reducing the AT profile, including the most harmful VAT and its subtype EAT [71,72]. However, it remains uncertain whether the potential benefits associated with reduction in EAT are independent of overall body weight reduction or simply reflect global metabolic improvement. Further mechanistic and imaging-based studies are needed to clarify the specific contribution of EAT reduction to HFpEF outcomes.

At the same time, another group of medications that has emerged as a cornerstone of HFpEF treatment is sodium-glucose transporter type 2 inhibitors (SGLT-2i). These drugs have shown remarkable results in the fields of diabetes, nephrology, and cardiology, including HF therapy regardless of ejection fraction, demonstrating beneficial effects for the patient in the spectrum of all three HF phenotypes, but most importantly in this context-in the treatment of HFpEF [63,73,74]. Although the mechanism of action of these medications is multifaceted and still under investigation, it is worth noting that the well-proven effect of osmotic diuresis caused by glucose excretion allows for a daily loss of up to 300 kcal [75]. This effect is associated with more favorable alterations of cellular energetic pathways, including cardiomyocytes [75]. More specifically, glucose loss favors the use of alternative energy fuels, where fatty acid oxidation may serve as an alternative and efficient energy substrate for myocardial cells [75]. Additionally, losing excess glucose and mobilizing other energy fuels is also associated with decreased production of advanced glycation end-products (AGEs) and concomitant reduced production of reactive oxygen species (ROS) [76,77]. As is well known, AGEs and ROS are very potent cell-damaging factors that can chronically mediate inflammation, in cardiac structures as well. Glucose excretion, reduction in ROS and AGE production, favorable metabolic alterations, and many more mechanisms during SGLT-2i intake allow for counteracting the pro-inflammatory effect of AT [76,77]. Accordingly, SGLT-2i may reduce adverse ventricular remodeling, preserve myocardial compliance, and prevent the increase in left ventricular filling pressures that underlie the development of symptomatic HFpEF [77,78]. Importantly, the interaction between AT and the NP system suggests that therapies which influence metabolic status may also indirectly decrease circulating NP levels [79,80]. Therefore, a comprehensive, phenotype-focused approach integrating weight management, pharmacotherapy, and careful interpretation of biomarkers appears essential to optimize treatment outcomes in patients with HFpEF and obesity. That is why future directions may include targeted therapies aimed at modulating AT inflammation, restoring NP signaling, and targeting metabolic factors in HFpEF [81]. These approaches may include the use of novel pharmacological agents targeting adipokine pathways, metabolic modulators, and strategies aimed at improving body composition rather than just body weight [81,82]. Furthermore, personalized, phenotype-focused treatment strategies integrating biomarker-based approaches may greatly improve diagnostic accuracy and therapeutic efficacy in patients with HFpEF and obesity [83]. A summary of the most important findings described in this work can be found in the summary Table 1.

## 9. Conclusions

AT is increasingly recognized as an important contributor to the pathophysiology of HFpEF, promoting inflammation, endothelial dysfunction, and adipokine-dependent cardiac remodeling. At the same time, increased body weight is associated with a paradoxical decrease in NP concentrations, most likely due to increased clearance in adipocytes and metabolic changes such as insulin resistance. This phenomenon may significantly impact the diagnostic process and prognosis of patients with a metabolic HFpEF phenotype. Additionally, the qualitative change in AT is also an important aspect—visceral fat appears to exert a more proinflammatory and metabolically adverse effect than SAT on the cardiovascular system. Additionally, it is worth noting that patients with HF and a low BMI, in contrast to individuals with obesity, demonstrate significantly higher NP concentrations, reflecting disease advancement and cachexia. Therefore, interpretation of NP concentrations should always take into account body composition and metabolic phenotype. Finally, therapies aimed at reducing body fat, which currently include GLP-1/GIP agonists and SGLT-2i, may represent promising therapeutic strategies for patients with the metabolic subtype of HFpEF.

This review has several limitations. As a narrative review, it is prone to potential selection bias. The inclusion of primarily recent studies (from 2020) may have resulted in the omission of previously relevant data. Furthermore, heterogeneity in the study population and methodology may limit the generalizability of conclusions.

## Figures and Tables

**Table 1:** Summary of the main findings and pathophysiological mechanisms described in the literature regarding the relationship between natriuretic peptides, adipose tissue, and heart failure with preserved ejection fraction.

Study	Main Findings	Clinical Significance
Packer et al. (JACC, 2025) [4]	Proposed the adipokine hypothesis linking dysfunctional adipose tissue with inflammation, endothelial dysfunction, and HFpEF development	Supports the mechanistic role of adipose tissue in metabolic HFpEF
Dronkers et al. (JACC, 2024) [1]	Demonstrated the complex relationship between obesity and HF progression	Highlighted obesity as a major contributor to HF pathophysiology
Peikert et al. (EHJ, 2025) [19]	Reported near-universal prevalence of central adiposity in HFpEF patients	Emphasized the importance of visceral adiposity over BMI alone
Janssen-Telders et al. (Cardiovasc Res, 2025) [16]	Described the role of epicardial adipose tissue remodeling in HFpEF	Suggested EAT as a potential therapeutic and diagnostic target
Van Woerden et al. (Circ Heart Fail, 2022) [36]	Showed association between increased EAT and worse HFpEF outcomes	Linked EAT accumulation with prognosis
Vaishnav et al. (JAHA, 2020) [8]	Observed lower NT-proBNP concentrations in patients with higher obesity class	Confirmed the obesity–natriuretic peptide paradox
Egom et al. (Front Physiol, 2021) [46]	Proposed NPR-C upregulation as a mechanism of increased NP clearance in obesity	Suggested a mechanistic explanation for reduced NP levels
Kozhuharov et al. (EJHF, 2022) [44]	Demonstrated the effect of obesity on NT-proBNP diagnostic cut-off values	Supported the need for obesity-adjusted NP thresholds
Selvaraj et al. (JACC Imaging, 2021) [61]	Linked lower muscle mass and altered body composition with elevated NP levels	Highlighted the contrast between obesity and cachexia phenotypes
Kosiborod et al. (NEJM, 2023) [64]	Demonstrated beneficial effects of semaglutide in obese HFpEF patients	Supported GLP-1 receptor agonists as a therapeutic option
Anker et al. (NEJM, 2021) [63]	Showed benefits of SGLT2 inhibitors in HFpEF	Established SGLT2 inhibitors as a cornerstone of HFpEF treatment

## Data Availability

No new data were created or analyzed in this study.
